# Three-dimensional-printed triply periodic minimal surface scaffolds via digital light processing for enhanced osteogenesis

**DOI:** 10.1093/rb/rbaf053

**Published:** 2025-06-05

**Authors:** Weilong Zou, Xiyuan Han, Qiyuan Dai, Zequ Lin, Qingtao Li, Zilin Li, Xinrong Xu, Xinying Chen, Huichang Gao, Xiaodong Cao

**Affiliations:** Department of Spinal surgery, Heyuan People's Hospital, Heyuan 517000, PR China; School of Materials Science and Engineering, South China University of Technology, Guangzhou 510641, PR China; School of Materials Science and Engineering, South China University of Technology, Guangzhou 510641, PR China; School of Materials Science and Engineering, South China University of Technology, Guangzhou 510641, PR China; School of Medicine, South China University of Technology, Guangzhou 510006, PR China; School of Materials Science and Engineering, South China University of Technology, Guangzhou 510641, PR China; School of Materials Science and Engineering, South China University of Technology, Guangzhou 510641, PR China; Department of Spinal surgery, Heyuan People's Hospital, Heyuan 517000, PR China; School of Medicine, South China University of Technology, Guangzhou 510006, PR China; School of Materials Science and Engineering, South China University of Technology, Guangzhou 510641, PR China; National Engineering Research Centre for Tissue Restoration and Reconstruction, South China University of Technology, Guangzhou 510006, PR China; Key Laboratory of Biomedical Engineering of Guangdong Province, South China University of Technology, Guangzhou 510006, PR China

**Keywords:** bioactive glass, DLP 3D printing, bone regeneration, TPMS structure

## Abstract

Natural bone is a naturally mineralized material with a nonhomogeneous porous structure, which is difficult to construct using conventional manufacturing methods. Triply periodic minimal surfaces (TPMS) have emerged as an excellent solution in recent years for constructing porous artificial bone structures, characterized by smooth surfaces, highly interconnected porous structures and mathematically controllable geometries. In this work, digital light processing (DLP) printing technology was used to construct a nonhomogeneous TPMS structure with strontium-doping 13-93 bioactive glass (Sr@BG) prepared by fusion method. The heterogeneous scaffolds were obtained by integrating high-strength I-wrapped package (I) and high-permeability Gyroid (G) units behaving a sufficient compressive strength of 5.8 ± 0.6 MPa, a porosity of ∼63% and a permeability of 0.97 × 10^−8^ m^2^, which matched the microstructural parameters of cancellous bone. Meanwhile, the biomimetic structure and Sr doping could cooperatively promote the adhesion, proliferation and differentiation of bone mesenchymal stem cells (BMSCs). In addition, the osteogenic ability of IG scaffolds was verified in rabbit’s femoral condylar defect. In general, heterogeneous IG scaffolds possess desirable bioactivity and mechanical property which meet the functional and structural requirements of bone regeneration.

## Graphical abstract

**Figure:**
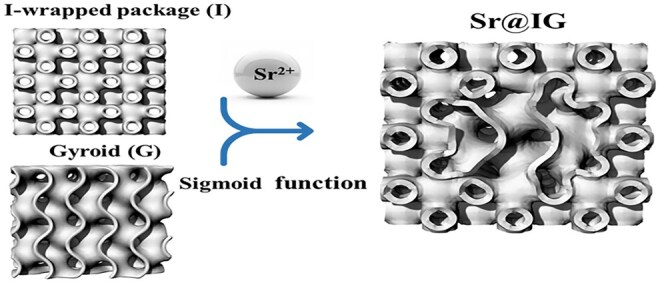

## Introduction

Traffic accidents, obesity and orthopedic diseases lead to an increase in bone fractures. Despite the current clinical use of many innovative therapies, there are still 10–20% of delayed or nonunion cases [1]. Autologous and allogeneic grafts are gold standards for most bone defects, while carrying the risks of donor deficiency and immunogenicity. In recent years, an increasing number of studies have focused on the development of artificial bone using porous biodegradable bioceramics [2]. Bioceramics are widely used for their osteoconductivity and mechanical compatibility with bone [3].

The structural design of porous scaffolds is a crucial factor to consider in tissue engineering scaffold construction [4]. Nonparametrically designed structures such as cubic cells [5], spatial polyhedral [6] and honeycomb [7], have been extensively explored regarding the mechanism, permeability and biological properties of scaffolds. However, nonparametrically designed scaffolds often risk such as strut thickness [8], interface mismatch [9], surface smoothness [10] and worse manufacturability due to the thermal deformation caused by long overhangs [11]. In addition, the straight edges or sharp turns of geometrically primitive’s shapes such as cylinders and cubes provide an unfavorable biomorphic environment for cell activities [12, 13]. Triply Periodic Minimal Surfaces (TPMS), a periodic implicit surface with a mean curvature of zero, parametric design and smooth connected surface, has shown the ability to reduce stress concentration, enhance mechanical strength and improve cellular activity [14–16]. Inadequate neovascularization may hinder the inward growth of implanted scaffolds, leading to failure of regeneration. TPMS has demonstrated the ability to promote osteogenic differentiation and angiogenic paracrine function in BMSCs by providing mechanical stimulation via a curved surface, enabling an osteogenic-angiogenic coupling [17]. Additionally, the surface of TPMS can disperse the stress and the mechanisms of various complex units are different [18].

Suitable permeability is crucial in constructing porous bone scaffolds to optimize nutrient transport [19]. Larger pore size or porosity improves permeability, directly impacting the proliferation and differentiation of multilineage cells [20, 21]. However, increased permeability often compromises mechanical strength. The use of different lattices to construct improved heterostructures has effectively addressed poor load-bearing capacity and insufficient permeability [22]. Thus, exploring the properties of units and heterogeneous TPMS may be an effective strategy to meet the structural needs of bone repair [23]. The structural features of scaffolds manufactured by common methods, such as polymer foam replication, freeze-drying and salt leaching, often lack precise control, resulting in poor mechanical strength, stress concentration and low porosity [24, 25]. DLP printing, with its high precision and printing efficiency, facilitates the production of porous scaffolds with connected pores and high complexity.

Bioactive glass (BGs) are essential inorganic biomaterials used for repairing hard tissues like bones and teeth, exhibiting strong osteogenic activity [26]. BGs are capable of binding strongly to soft and hard tissues in body fluids by forming hydroxyl-carbonate-apatite (HCA) layers after ion release. However, BGs with low Si content, such as 45S5 or ICIE16 BGs, behave a narrow sintering window and are prone to crystallization during the sintering process, leading to impaired mechanical strength and bioactivity [27]. The development of 13-93 BG with a larger sintering window, achieved by increasing Si content and decreasing Na_2_O content partially replaced by K_2_O and MgO, has significantly improved sintering performance [28]. The 13-93 BG scaffold exhibits favorable mechanical properties, making it an ideal choice for bone tissue engineering scaffolds [29]. In addition, the doping of various therapeutic ions is gradually becoming an effective selection for the treatment of bone defects [30–32].

**Figure 1.**
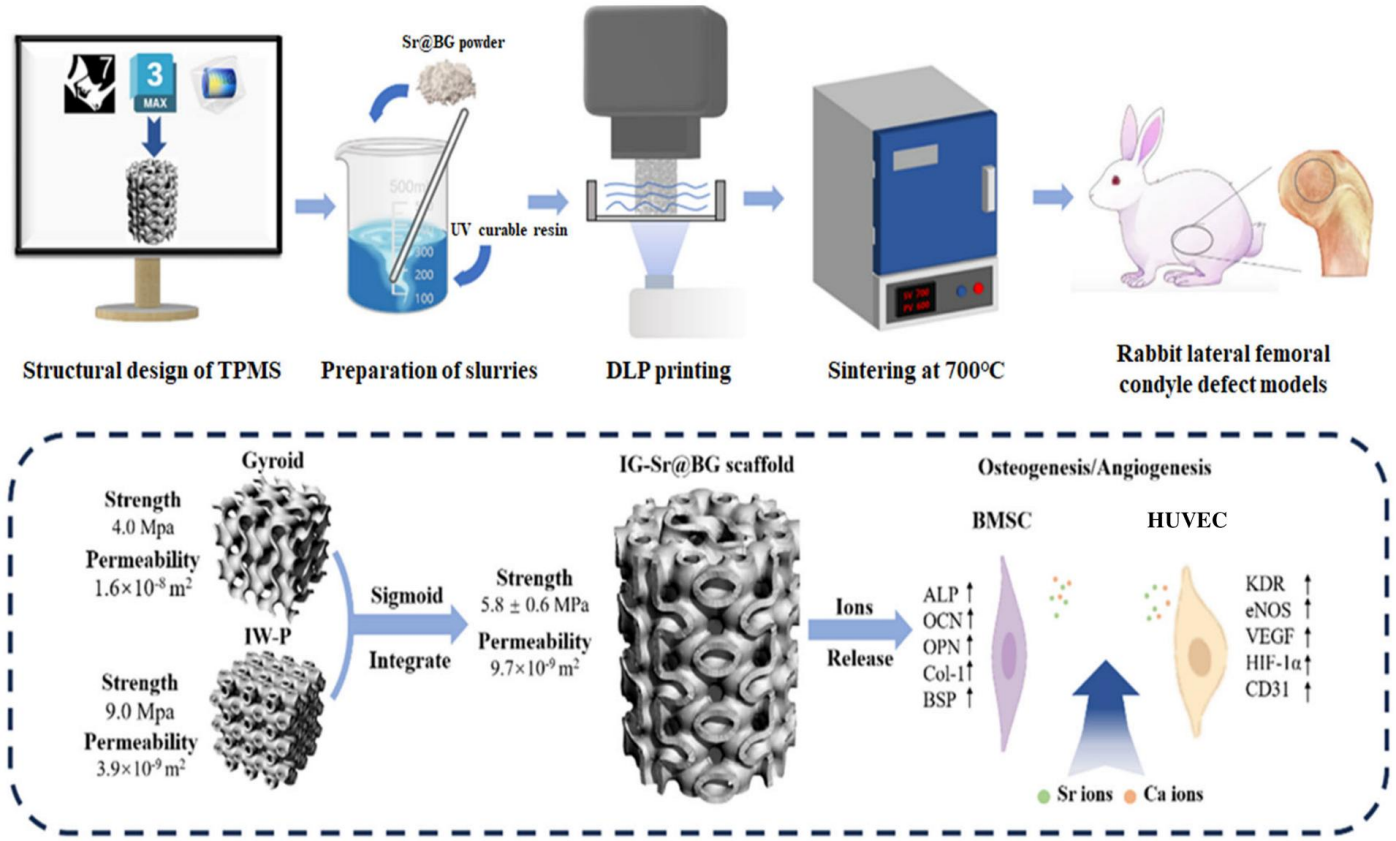
Illustration of fabrication and applying process of Sr@IG scaffolds.

In this study, BG scaffolds were fabricated with optimized porosity, mechanical strength and permeability by blending the selected units, I-Wrapped Package (I) and Gyroid (G) and enabling the formation of heterogeneous scaffolds (IG) through the application of a sigmoid function. The resultant IG scaffold demonstrated simultaneous compressive strength and permeability, thereby promoting cell proliferation and differentiation. *In vivo* experiments indicated that both the scaffold architecture and addition of a small amount of Sr significantly facilitated bone regeneration (Figure 1). This methodology presents a viable strategy for the customization of artificial bone scaffolds with tailored structures, ultimately enhancing their bioactivity.

## Materials and methods

### Preparations of Sr@BGs powders

Analytical grade SiO_2_, CaCO_3_, NaH_2_PO_4_, K_2_CO_3_, Na_2_CO_3_, MgO and SrCO_3_ were purchased from Aladdin Chemistry (Shanghai, China). Ethanol was supplied from Guangzhou Chemical Reagent and Factory (Guangzhou, China). Three-dimensional Printer UV curable resin was obtained from CREALITY (Shenzhen, China). Briefly, the above raw materials were mixed well according to the proportions in Supplementary Table S1 and transferred to an Al_2_O_3_ crucible. Then, the powders were heated up to 1400°C for 2 h. The crucible was quickly removed, and the melt was quenched by pouring it into ice-cold deionized water to obtain Sr@BGs frits. All Sr@BGs frits were ground in a centrifugal ball mill (QM-1SP2CL, Nanda, China) for 4 h with ethanol as solvent. Finally, the dried powders were gently pressed to allow them to pass through a stainless-steel mesh with a pore size of 40 μm. As 0, 2.0, 5.0, 10.0 and 22.0 mol% of CaO in BGs were replaced by SrO, the samples were named as 0Sr@BG, 2Sr@BG, 5Sr@BG, 10Sr@BG and 22Sr@BG, respectively.

### Preparation of TMS-based BG scaffold

TPMS-based scaffolds were built with Rhino 7 by applying different formulas. To construct a 3D surface with smooth transition, each point will be considered as the smallest cubic unit in a spatial array, TPMS function was applied the to the cubic array of points, and closest planes were extracted to the function while removing the noncontacting planes. The pore sizes of the scaffolds were selected to be 1000, 700 and 500 μm. The models of crosshatch, Gyroid, Schwarz P, Diamond and I-Wrapped Package were fabricated, which were represented by C, G, P, D and I, respectively. Meanwhile, taking the C structure as an example, the scaffolds with pore sizes of 1000, 700 and 500 μm were named C1000, C700 and C500. The construction of the heterogeneous scaffolds involved the sigmoid function, which facilitated the rapid transition between multiple types of units and realized structural integration, and its basic form was given in the following equation:


(1) f(x)=11+e-x.

The functions of integrating two TPMS units were shown in the following equations:


(2) S12=α(x,y,z)·S1+(1-α(x,y,z))·S2,

(3) α(x,y,z)=11+e-k·G(x,y,z),

where *S_i_*—different TPMS equations, *α*(*x, y, z*)—weighting factor, *k*—gradient factor, *G*(*x, y, z*)—Inter-unit junction equation. Larger values of *k* indicate narrower transition zones between units, and *G*(*x, y, z*) determines where the units integrate.

The 3D printing ink was prepared by mixing the Sr@BG powders with UV curable resin at room temperature to achieve a weight ratio of 50 wt%. Subsequently, the ink is transferred to the liquid tank integrated into the DLP printer (Bio-architect^®^ PR, Regenovo). After calibration, the designed models were imported and the scaffolds in cube shapes (10 × 10 × 10 mm) were printed out and sintered at 700°C for 2 h to be densified. The thermogravimetric curves of printed scaffolds were obtained with thermogravimetric analysis (TG-DSC, Snetzsch, Germany) at a rate of 5°C/min up to 800°C. The morphology and composition analysis of Sr@BGs were initially performed using a field emission scanning electron microscope (SEM, Merlin, Zeiss, Germany) equipped with energy-dispersive spectroscopy (EDS). Furthermore, X-ray diffraction (XRD, BRUKER AXS GMBH, Germany) and Fourier-transform infrared spectroscopy (FTIR, IS50, Thermo Fisher, USA) techniques were employed to investigate changes in crystalline phase and chemical structure. Five different pore size structures of Sr@BGs scaffolds were fabricated, and their morphologies were characterized by SEM. The scaffold samples had dimensions of 7 × 7 × 7 mm cubes which underwent compression testing on a universal material testing machine (5697, Instron, USA), applying a load of 30 kN at a loading rate of 1 mm/min. The largest compressive stress and strength were recorded during compression, and at least three samples were tested. In addition, the porosity was measured by Archimedes drainage method. The sample was first weighed in air, which was recorded as m_0_. The scaffold was then submerged in anhydrous ethanol under vacuum for 60 min and placed in a specific gravity balance filled with ethanol to obtain the mass of the scaffold in anhydrous ethanol noted as m_1_. Finally, the scaffold was taken out and measured its wet weight in air which was recorded as m_2_. The porosity of the scaffold was calculated according to the equation:


$$P=\frac{\left(m_{2}-m_{0}\right)}{\left(m_{2}-m_{1}\right)}\times 100\%.$$

### HAp formation ability of Sr@BGs

The BGs were transferred into polyethylene bottles filled with simulated body fluids (SBF) (Supplementary Table S2) at a concentration of 1 mg/mL and placed in a constant temperature shaker at 37°C with 120 rpm for 7 days. Then, the Sr@BGs powders were collected, and the SEM was used to observe their surface morphology. Meanwhile, the XRD and FTIR analyses were performed to determine the changes in the glass crystalline phase and chemical structure at 1, 3 and 7 days. In addition, the concentrations of ions released from the BGs were determined by inductively coupled plasma emission spectroscopy (iCAP 7200 Duo). Similarly, to observe the HAp formation of the sintered BGs scaffolds, the same experiments were performed on the sintered Sr@BGs discs.

### Finite element simulation analysis

During the analysis process, incompressible steady-state laminar Navier–Stokes with constant density and viscosity were used. The equation is:


$$\rho \frac{\partial u}{\partial t}-\mu \nabla^{2}u+\varphi (u,\nabla )u+\nabla P=F,\nabla \cdot u=0,$$

*ρ* represented the fluid density, kg/m^3^,

*μ* represented the Dynamic viscosity of the fluid, kg/m·s,

u represented the Fluid flow velocity, m/s,

∇ represented the del operator,

*P* represented the Pressure, Pa,

*F* represented the Gravity or centrifugal force,

*ρ* represented the fluid density, kg/m^3^,

*μ* represented the Dynamic viscosity of the fluid, kg/m·s,

*u* represented the Fluid flow velocity, m/s,

∇ represented the del operator,

*P* represented the Pressure, Pa,

*F* represented the Gravity or centrifugal force.

Finite element analysis was performed using COMSOL MultiPhysith 6.1 to explore the permeability of different structures. Briefly, permeability was calculated by setting the viscosity (*μ*) to 0.001 pa·s, the inlet velocity to 0.1 mm/s and the boundary pressure at the outlet to 0. The rest of the surfaces were set to be no-slip surfaces. Permeability was calculated according to the following equation:


(4) k=QAμLΔP,

where Q*—*volume flow rate (m^3^/s), *L—*length between inlet and outlet (m), *A—*area of the flow supply (m^2^) and ΔP—pressure difference between the inlet and outlet pressures (MPa).

COMSOL simulation analysis process: The scaffold in different groups was imported into 3D MAX. Boolean operations were performed on the scaffold using a cube of the same size. The corresponding fluid area could be obtained and saved in STL format. The fluid region model was imported into COMSOL for setting boundary conditions, meshing and calculations. The specific boundary conditions were set as follows: (1) The inlet flow velocity was set as 0.01 m/s and the outlet pressure was set as 0 Pa. (2) There is no sliding on the wall surface. (3) The properties of the fluid were set as water flow, that is, the fluid density was 1000 kg/m³ and the fluid dynamic viscosity was 0.001 Pa·s.

WSS Simulation: The wall shear stress (WSS) was also analyzed. The dynamic viscosity of the fluid was 0.0037 Pa·s (cell culture medium containing 5% wt/wt dextran) according to previous research [33]. WSS was calculated according to the following equation:


(5) τω=μ∂u∂h,

where *u* denoted the flow velocity and *h* represented the *x*-, *y*- and *z*-directions.

### Osteogenic activities of Sr@BGs powders and Sr@BGs scaffolds

The osteogenic abilities of Sr@BGs powders and Sr@BGs scaffolds were further investigated by *in vitro* cell experiments. For culturing the bone mesenchymal stem cells (BMSCs), 0.2 g/mL of DMEM extracts were used and diluted to 1 mg/mL with complete medium containing 10% (v/v) fetal bovine serum (FBS, Gibco, USA), 100 U/mL penicillin and 0.1 mg/mL streptomycin. Human umbilical vein endothelial cells (HUVECs) (HUVEC-20001) were purchased from Cyagen Biosciences (Guangdong, China). BMSCs (CRL-12424) were purchased from the American Type Culture Collection (ATCC, USA).

The effect of Sr@BGs powders on cell proliferation was firstly investigated. Briefly, BMSCs were seeded on a 48-well plate with 5 × 10^3^ cells per well and cultured for 24 h. After that, the culture medium was replaced by Sr@BGs powders extracts and refreshed every other day. Cell counting kit-8 assay (CCK-8, Japan) was applied to investigate the effect of extracts on cell proliferation at day 1, 3 and 5. The OD values at 450 nm were detected by a microplate reader (Varioskan Flash 3001, Thermo Fisher, USA). Cells cultured in the complete medium were used as control group.

The osteogenic abilities of Sr@BGs powders and Sr@BGs scaffolds were further investigated. In brief, BMSCs were inoculated in 48-well plates at a density of 1 × 10^4^ cells/per well and incubated with Sr@BGs powders extracts supplemented with 0.25 mM ascorbic acid, 10 nM β-glycerophosphate and 20 nM dexamethasone. At each time point (7 and 14 days), BCIP/NBT Alkaline Phosphatase (ALP) Chromogenic Kit (Beyotime, China) was performed to characterize ALP expression. Meanwhile, ALP activity of BMSCs was also determined by ALP assay kit (Beyotime, China) after lysing with RIPA buffer (Beyotime, China). In addition, BMSCs incubated with extracts for 7 days were stained with 2% (w/v) Alizarin Red Solution (pH 4.2) for 30 min and observed by light microscope to assess extracellular matrix mineralization. Furthermore, the relative expressions of osteogenic related genes were quantified by reverse transcription-polymerase chain reaction (RT-PCR). Briefly, BMSCs were seeded into 48-well plates at 1 × 10^4^ cells/well and incubated with extracts for 7 and 14 days. At each time point, total RNA from BMSCs was extracted using the HiPure Total RNA Micro Kit (Magen, China) according to the method provided by the manufacturer. Then, the RNA was transcribed by the PrimeScript RT kit (Takara, Tokyo, Japan) to obtain complementary DNA (cDNA). Finally, the gene expression levels of osteocalcin (OCN), osteopontin (OPN), Bone sialoprotein (BSP) and collagen I (Col-I) were detected using RT-PCR (QuantStudio 6, Thermo Fisher, USA).

BMSCs were then inoculated on the scaffolds at a density of 1 × 10^4^ cells/well in a 48-well plate. The cell proliferation was determined at 1, 3 and 7 days. To characterize the cell viability on each group, BMSCs were seeded on the scaffolds (7 × 7 × 3 mm) with 2 × 10^4^ cells per well. The cell viability was determined by live/dead staining kit at 1 and 3 days, respectively. Furthermore, BMSCs were seeded on different structural scaffolds at a density of 1 × 10^4^ cells/well in a 48-well plate and the expression of the osteogenic genes was determined at 7 days.

### Angiogenic activities of Sr@BGs powders

The angiogenic abilities of Sr@BGs powders were further investigated by *in vitro* cell experiments. For culturing HUVECs, ECM containing 1% (v/v) of endothelial cell growth supplement (ECGS) were used to dilute extracts to 1 mg/mL. All cells were incubated in a humidified incubator (37°C, 5% CO_2_).

The ability of cell recruitment in HUVECs was tested with Sr@BGs powders extracts. Briefly, 5 × 10^4^ cells (200 μl cell suspension) were seeded into the upper wells of 24-well plate and 600 μl of extracts were placed into the bottom wells. After 12 h of incubation, the cells were fixed with 4% paraformaldehyde and then stained with 0.1% crystal violet for 30 min. Cells recruited from the upper chamber to the bottom chamber were observed using a light microscope. Typical images of each group were recorded, and five areas of 200 × 200 μm were selected randomly for cell counting.

In addition, immunofluorescence staining was used to observe the expression of CD31. CD31, also known as platelet endothelial cell adhesion molecule-1 (PECAM-1), is a transmembrane glycoprotein expressed predominantly on endothelial cells on the inner surface of blood vessels. It plays a crucial role in cell adhesion, migration and angiogenesis. In brief, HUVECs were seeded on laser confocal dishes at a density of 5 × 10^4^ cells/0.2 mL. After overnight incubation, extracts were added and a three-day cell culture started. Then, the cells were sequentially treated with 4% paraformaldehyde, 0.1% Triton X-100 (BioFroxx), CD31 primary antibody (1:500 dilution, Abcam, England) and donkey anti-rabbit IgG cross-adsorbed secondary antibody Alexa Fluor^®^ 555 coupling (1:500, Beyotime, China). The nuclei were stained by incubating with DAPI (C1002, Beyotime, China) for 10 min, and then, washed with PBS. Finally, the samples were observed under a laser scanning confocal microscope (TCS SP8, Leica, Germany).

For angiogenic genes, the total RNA in HUVECs was extracted after 7 days of incubation with extracts and transcribed into cDNA. The gene expression levels of vascular endothelial growth factor (VEGF), VEGF receptor 2 (KDR), hypoxia-inducible factor-α (HIF-α) and endothelial nitric oxide synthase (eNOS) in HUVECs were assessed by RT-PCR. Glyceraldehyde-3-phosphate dehydrogenase (GAPDH) was selected as the housekeeping gene for all target genes, and the relative quantifications of the genes were normalized by GAPDH and calculated by the 2^-ΔΔCΤ^ method. All primer sequences were shown in Supplementary Table S3, and each group was tested at least three times.

### *In vivo* bone repair in rabbit lateral femoral condyle defect models

Three-month-old male New Zealand white rabbits weighing 1.5–2.0 kg were chosen for followed surgery. The procedure was performed by drilling a cylindrical defect at 6 mm in diameter and 8 mm in depth on the lateral femoral condyle. Sixteen rabbits were equally divided into blank defect group, C (cross-hatch) group, IG group and Sr@IG group. Each scaffold matching the defect was then implanted. The blank group take no scaffold. Animal Ethics Committee approved all animal procedures and treatment of South China University of Technology (CV2020001).

At 4 and 12 weeks after surgery, the rabbits were sacrificed to obtain intact rabbit femurs. The samples were then scanned and reconstructed in Micro-CT (PerkinElmer Quantum GX2) at a spatial resolution of 24 μm. All data were reconstructed and visualized using Materialise Mimics Research (v 21.0) with a uniform minimum threshold of 1200. Finally, new bone volume/total volume (BV/TV), bone mineral density (BMD) and trabecular thickness (Tb.Th) were defined and calculated in CTAn. Each sample was embedded in methyl methacrylate and then sectioned of 10 μm thickness. Hard tissue sectioning were stained with Methylene Blue/Basic Fuchsin and Van Kossa for subsequent histological analysis.

### Statistical analysis

All quantitative data were depicted as mean ± standard deviation. A one-way ANOVA followed by a Tukey test for means comparison was performed to assess the level of significance. The value of *P* < 0.05 was considered to be statistically significant. The *, ** and *** represented a significant level at 0.05, 0.01 and 0.001, respectively. All experiments were conducted at least 3 times to guarantee the reproducibility of the findings.

## Results

### Morphology and *in vitro* bioactivity of Sr@BGs powders

In the preparation process of BGs, a series of Sr@BGs powders with different Sr content were prepared by replacing CaO in BGs with 0, 2.0, 5.0, 10.0 and 22.0 mol% of SrO. They were named 0Sr@BG, 2Sr@BG, 5Sr@BG, 10Sr@BG and 22Sr@BG. The morphology and structure of Sr@BGs powders with different Sr content was firstly investigated and the results were shown in Supplementary Figure S1. The SEM results found that the powders presented irregular particles with size basically below 10 μm after sieving (Supplementary Figure S1A). In addition, XRD and FTIR were used to characterize the structure of the Sr@BGs and the results were shown in Supplementary Figure S1B and C. All groups of Sr@BGs exhibited amorphous broad bands between 20 and 30° and there was no crystallization in XRD pattern. At the same time, the characteristic bands of silicate glass appeared in FTIR as Si-O-Si bending vibration at 500 cm^−1^ and Si-O-Si asymmetric stretching vibration at 900–1200 cm^−1^ [34].

**Figure 2.**
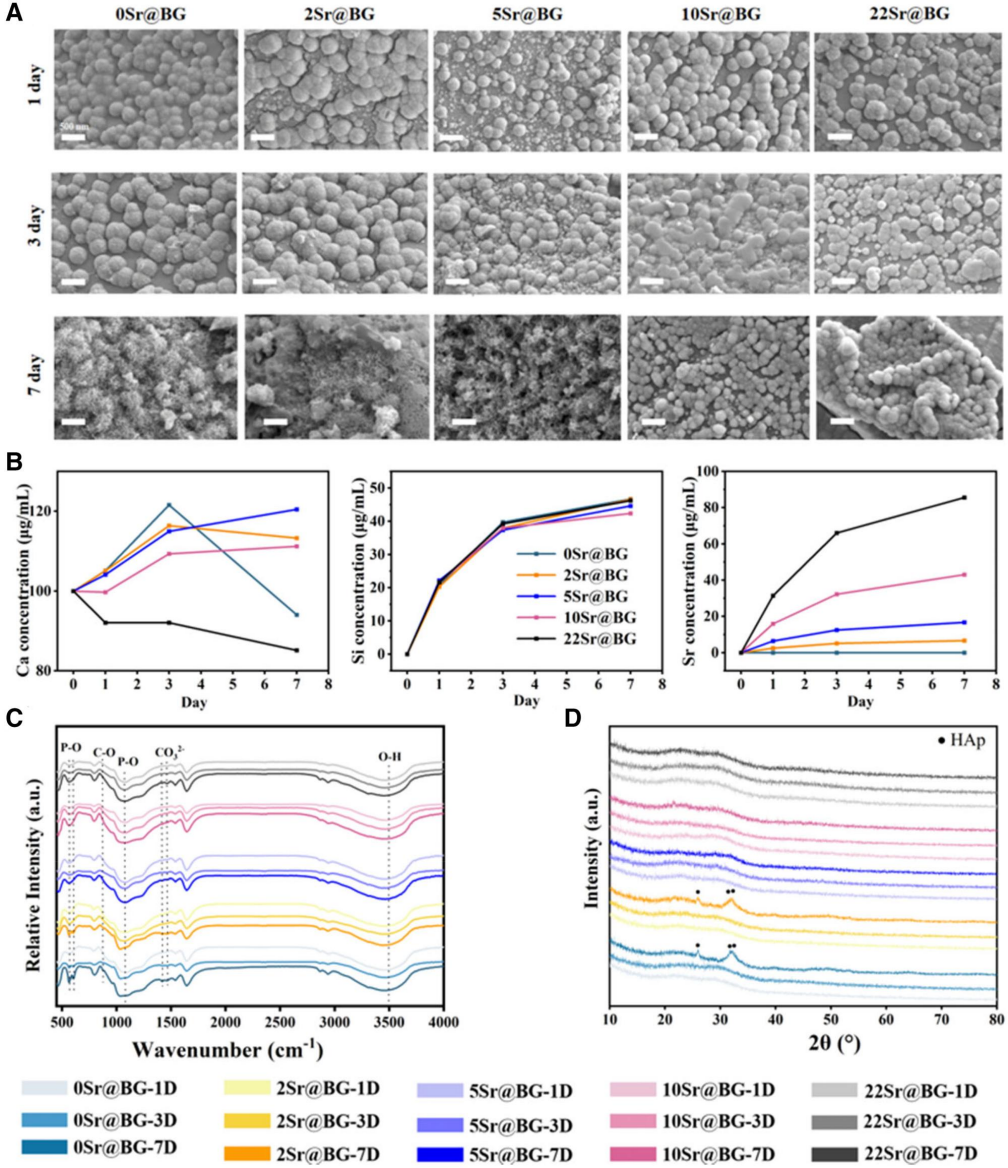
*In vitro* mineralization of Sr@BGs powders with different Sr content. (**A**) SEM images of Sr@BGs powders with different Sr content after mineralization *in vitro*. (**B**) The release curve of Ca, Si and Sr in the SBF at each time point during the mineralization. (**C)** FTIR and (**D**) XRD characterization of Sr@BGs powders after mineralization *in vitro*.

The ability of Sr@BGs to form hydroxyapatite (HAp) was further assessed by soaking it in SBF for 1, 3 and 7 days, and the results were shown in Figure 2A. It was found that increasing deposits of amorphous calcium phosphate microspheres appeared on the surface of all groups in the early stage. Both 0Sr@BG and 2Sr@BG showed significant mineralization of HAp at 7 days, while Sr@BGs with higher Sr content remained unmineralized. The release of Ca, Si, Sr ions from Sr@BGs powders in SBF was examined. As shown in Figure 2B, the content of Ca in 2Sr@BG and 0Sr@BG continued to increase for 3 days. However, Ca concentration decreased sharply at 7 days, indicating that mineralization dominated the change of Ca concentration, and a large amount of Ca was consumed to form HAp. Besides, the release of Sr increased with the increase of Sr content in Sr@BGs, which may be the main factor affecting the formation of HAp, because the high concentration of Sr locally competed for the nucleation position of Ca, preventing crystallization. In addition, Si was not consumed in this process, thus, maintaining a continuous release until the balance of mineralization and degradation was reached. Meanwhile, XRD and FTIR characterization of Sr@BGs powders after mineralization *in vitro* were carried out. As shown in Figure 2C and D, 0Sr@BG and 2Sr@BG powders at 7 days possessed apparent crystallization peaks of HAp. The P-O bending vibrational peak and stretching peak of the hydroxyl group (3500 cm^−1^) became sharper with the increasing of soaking time. The peaks at 873 cm^−1^ corresponded to the vibrational modes of C-O bonds in hydroxycarbonate apatite (HCA). Meanwhile, the peaks at 1420 cm^−1^ and 1455 cm^−1^ indicated the presence of ${\text{CO}}_{3}^{2-}$. All these phenomena indicated that a large amount of HCA was formed on the surfaces of 0Sr@BG and 2Sr@BG.

### Cytocompatibility of Sr@BGs powders

**Figure 3.**
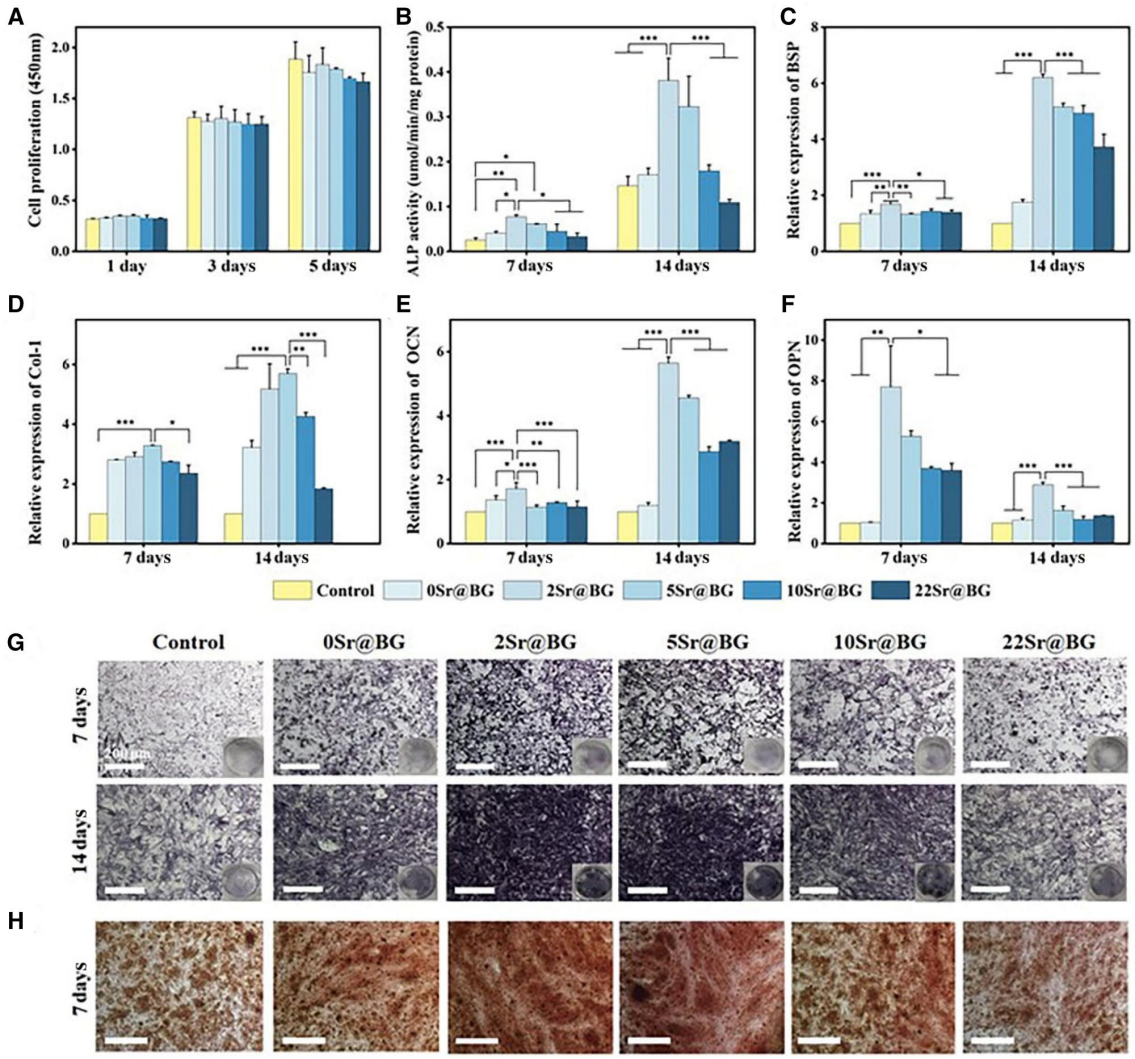
Effects of Sr@BGs powders extracts on osteogenic differentiation of BMSCs. (**A**) Cell proliferation of BMSCs incubated with all Sr@BGs groups for 5 days. (**B**) Quantitative analysis of ALP activity in BMSCs after culturing with extracts of different groups for 7 and 14 days. The expression of osteogenesis-related genes, including (**C**) BSP, (**D**) Col-I, (**E**) OCN and (**F**) OPN. (**G**) Images of ALP staining after culturing BMSCs with each extract for 7 and 14 days. (**H**) Images of alizarin red staining after culturing BMSCs with each extract for 7 days (*P < 0.05, **P < 0.01, ***P < 0.001).

The cytocompatibility of the Sr@BGs with different Sr content was assessed by cell proliferation experiment. As shown in Figure 3A, all groups of Sr@BGs powders extracts were able to support the normal proliferation of BMSCs, indicating that Sr@BGs powders with different Sr ion content possessed favorable cytocompatibility.

### Osteogenesis of Sr@BGs powders

The ability of Sr@BGs powders to promote osteogenesis was evaluated through ALP expression, Alizarin Red S (ARS) staining and the expression of osteogenesis-related genes. The results of ALP staining and quantification assays were shown in Figure 3B and G. It was found that deeper staining in the 0Sr@BG group compared to the control group, suggesting that Ca and Si ions released from BG contributed to enhance osteogenic capacity of BMSCs. Sr ion doping further increased ALP expression, with 2Sr@BG exhibiting the strongest staining. However, higher Sr content gradually decreased the expression of ALP. In addition, the RT-PCR results in Figure 3C–F showed that the gene expressions of OCN, OPN and BSP in 2Sr@BG group were evidently higher than other groups, whereas the expression of Col-I was slightly lower than 5Sr@BG. Apparently, a small amount of Sr played an important role in contributing to osteogenic differentiation, with the optimal effect observed with 2Sr@BG powder extract. Additionally, the mineralization of the extracellular matrix of BMSCs was assessed through ARS staining. As shown in Figure 3H, the qualitative results of calcium nodules revealed that Sr@BGs with lower Sr content exhibited increased mineralization and osteogenic differentiation of BMSCs, as evidenced by more prominent red-stained calcium nodules.

### Angiogenesis of Sr@BGs powders

**Figure 4.**
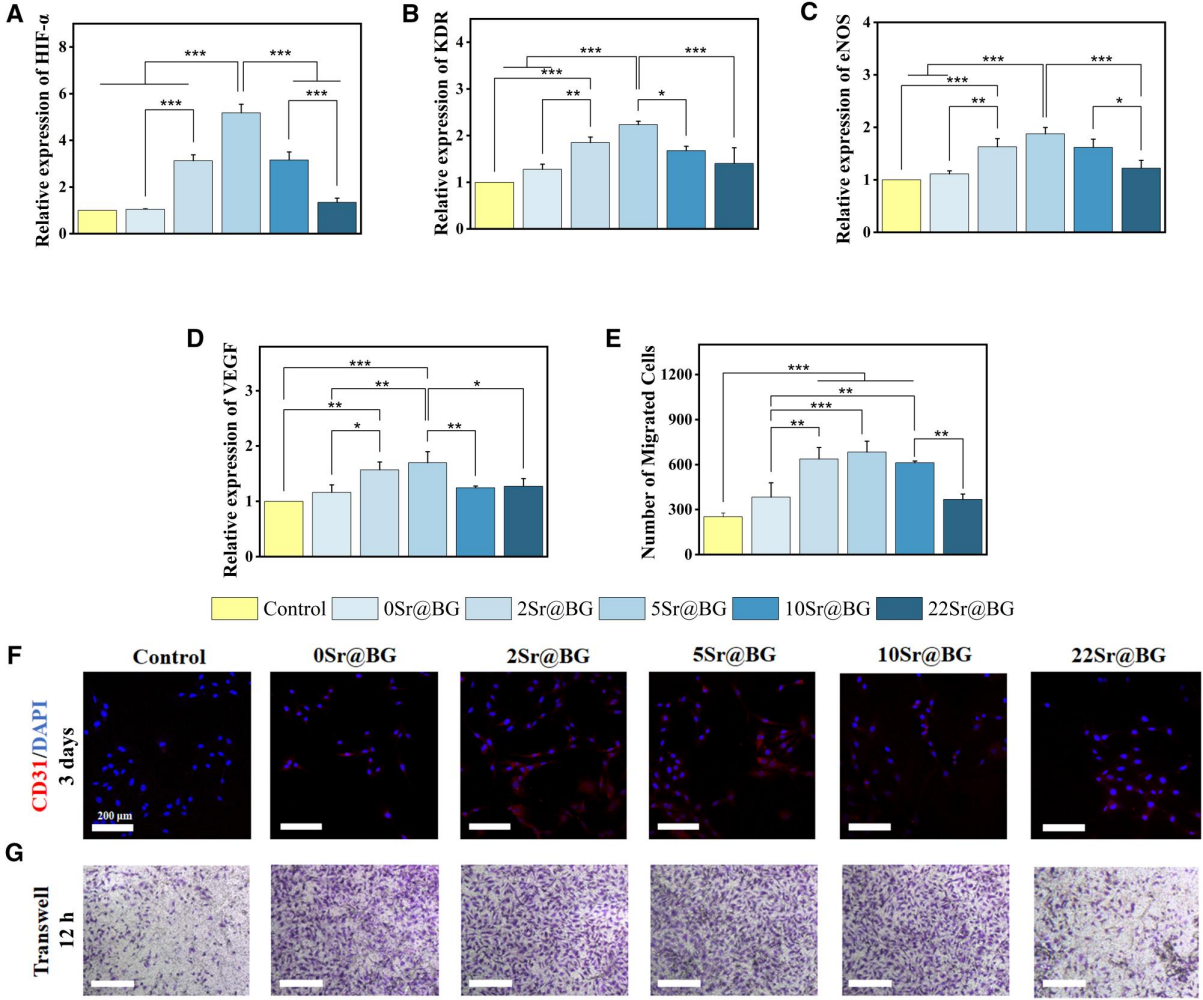
Effect of Sr@BGs powders extracts on the angiogenesis of HUVECs. The expression analysis of (**A**) HIF-α, (**B**) KDR, (**C**) eNOS and (**D**) VEGF genes in HUVECs after culturing with the extracts for 7 days. (**E**) Quantitative analysis of cell number in an area of 200 × 200 μm. (**F**) Immunofluorescence staining of CD31 and DAPI in HUVECs. (**G**) Images of crystal violet staining for transwell migration assay (*P < 0.05, **P < 0.01, ***P < 0.001).

The formation of blood vessels is critical in providing nutrients and discharging wastes during bone regeneration. Therefore, the angiogenic ability of Sr@BGs powders extracts was verified. First, the expressions of angiogenesis-related genes (HIF-α, KDR, eNOS and VEGF) were determined by RT-PCR. As shown in Figure 4A–D, the results showed that after 7 days of incubation, the expressions of all genes in HUVECs exhibited an increasing and then decreasing trend with the rising of Sr content, with the highest levels observed in the 5Sr@BG group. Furthermore, immunofluorescence staining experiments were taken to determine the expression of marker proteins in HUVECs during angiogenesis. Red and blue colors represented the staining of CD31 and nucleus, respectively. As shown in Figure 4F, the results were consistent with the trend of gene expression. In addition, Transwell experiments also demonstrated the pro-migratory effect of the extracts on HUVECs to different degrees (Figure 4E and G). Therefore, these results demonstrated the groups with low content of Sr possessed a better angiogenic performance.

### Preparation and characterization of TPMS-based BG scaffolds

**Figure 5.**
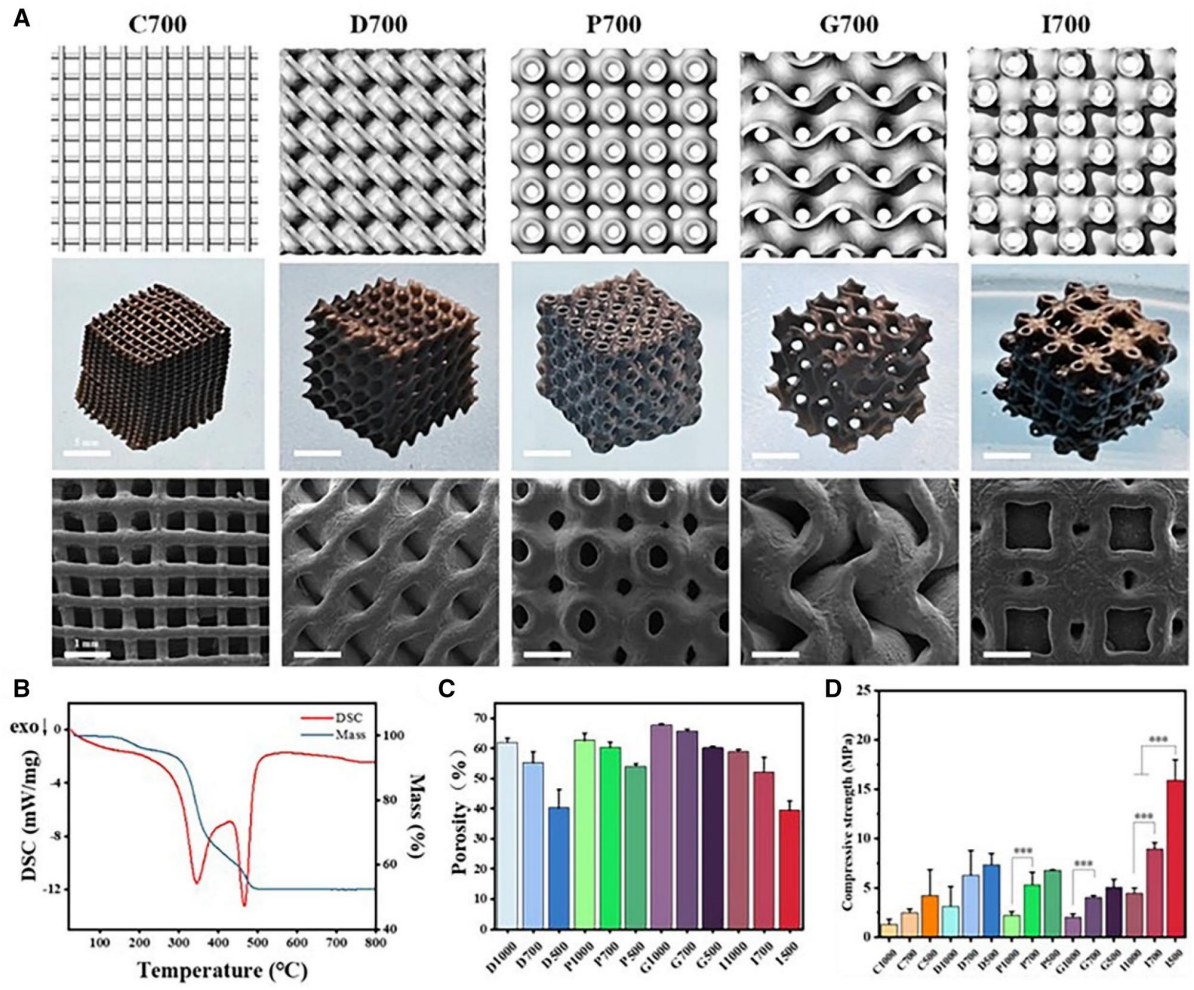
Physical characterization of different structural BG scaffolds. (**A**) Modeling and the morphology of BG scaffolds with different structures of 700 μm pore size. (**B**) TG-DSC analysis of printed scaffolds. Characterization of (**C**) porosity and (**D**) compressive strength of different structural BG scaffolds with different pore sizes (1000, 700, 500 μm) (*P < 0.05, **P < 0.01, ***P < 0.001).

Models of TPMS were produced in Rhino 7 by adjusting the period parameter in the formulae to change the pore size of the scaffolds (1000, 700 and 500 μm), with a wall thickness of 300 μm and dimension of 10 × 10 × 10 mm, as depicted in Supplementary Figure S2. The morphology of the scaffolds with a pore size of 700 μm after sintering was shown in Figure 5A. The shrinkage of the scaffolds was uniform and around 35%. The surfaces were connected smoothly after sintering, and the shapes could be well maintained. The TG-DSC presented the changes of the scaffolds during the sintering process. As shown in Figure 5B, it was found that two obvious exothermic peaks before 500°C, and the mass was significantly reduced to 52.8 wt%, which was the process of excluding the organic matter, indicating that the organic matter was basically removed during the sintering process.

Physical characterizations, including porosity and compressive strength, were performed. Porosity was tested by the saturated alcohol method, as shown in Figure 5C. The porosity of the scaffolds was in the range of 40–70%, and the corresponding porosity of the scaffolds reduced with the decrease of pore size. In addition, there was an obvious tendency for compressive strength to improve with smaller pore size (Figure 5D), because the increase of porosity tends to concentrate stress and destroy the mechanical properties of the scaffolds, the compressive modulus also shows a similar trend (Supplementary Figure S6). The strength of the scaffolds was in the range of 1.3–15.89 MPa, which could basically match the requirement of cancellous bone (2–12 MPa). It was noteworthy that all the TPMS exhibited better mechanical strength than that of C, which should be affected by the high stress concentration at the cross nodes. Furthermore, the I unit scaffold possessed the highest mechanical strength (4.4–15.9 MPa), while the G behaved the worst (2.0–5.2 MPa). The ranking of mechanical properties in each structure are C (2.5 MPa) < G (4.0 MPa) < P (5.3 MPa) < D (6.3 MPa) < I (9.0 MPa). C and G exhibited the lowest compressive strength, due to high stress concentration at the nodes where the pillars cross and overlap in the structure of C, while G behave a higher porosity and a surface with high stress concentration.

### Design and evaluation of heterogeneous scaffolds

**Figure 6.**
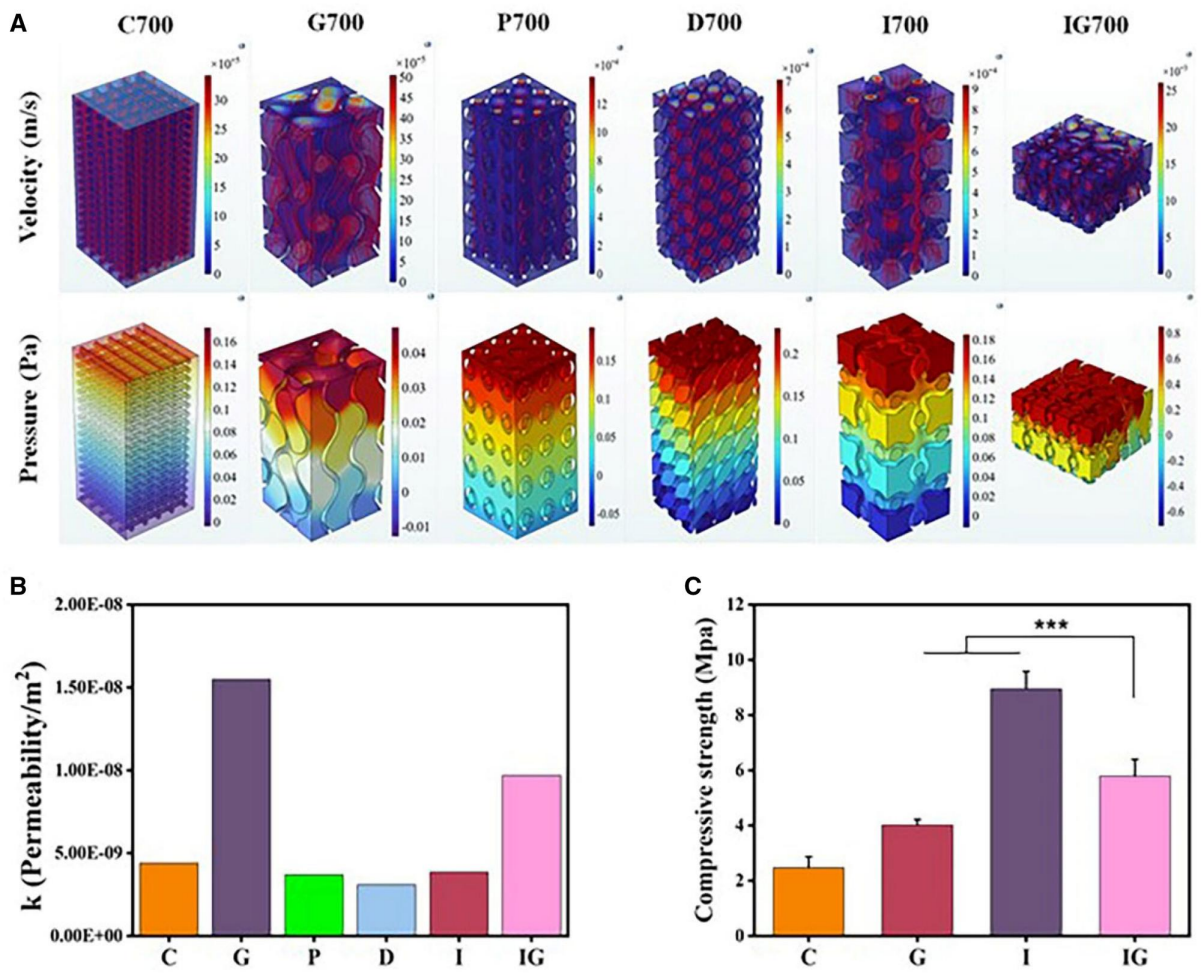
Design of heterogeneous structures and optimization of performances. (**A**) Permeability analysis of different structural scaffolds, including both velocity and pressure cloud diagrams. (**B**) Quantitative analysis of permeability of different structural scaffolds. (**C**) Mechanical strength of different structural scaffolds with a pore size of 700 μm (*P < 0.05, **P < 0.01, ***P < 0.001).

**Figure 7.**
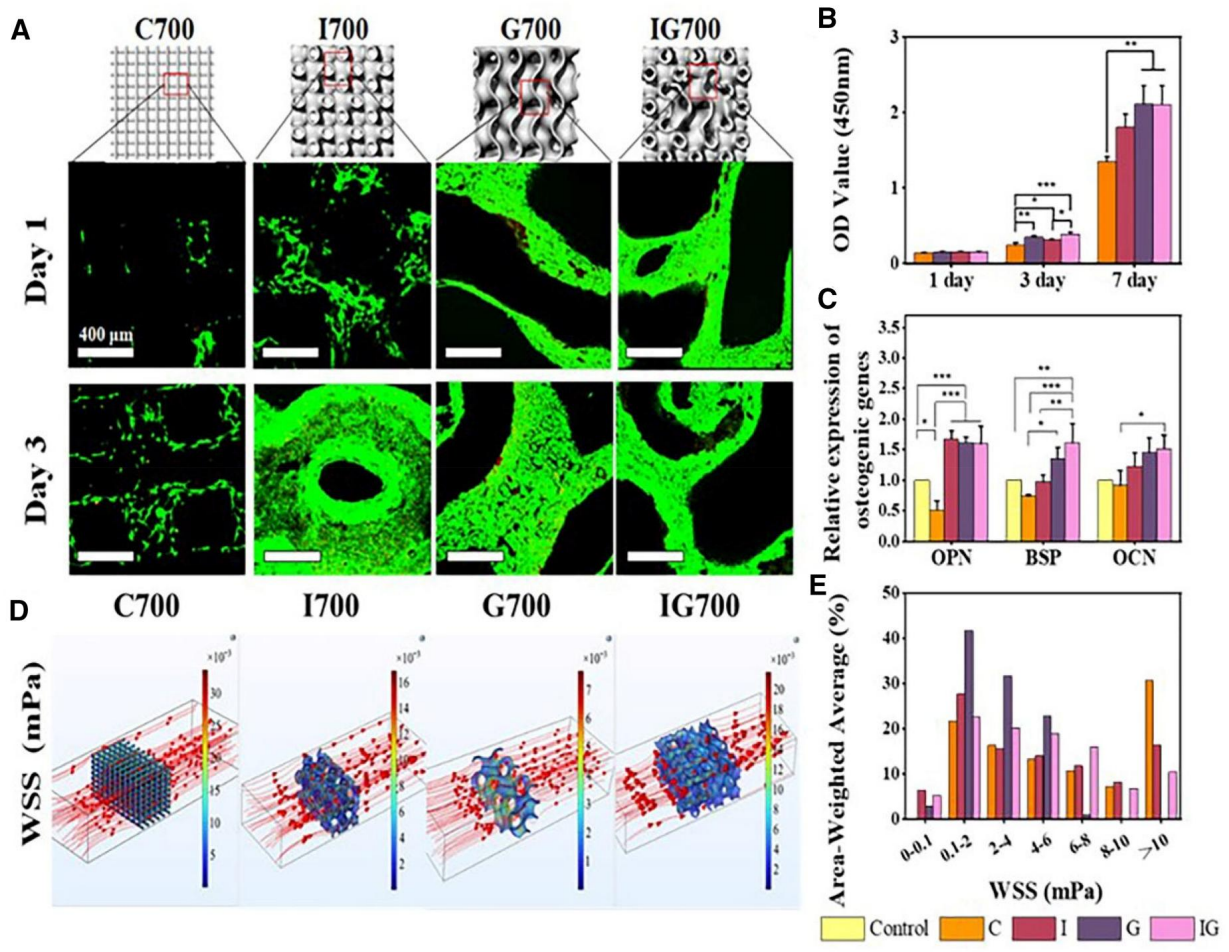
Evaluation of the biological properties of different structural scaffolds. (**A**) Live-dead staining and (**B**) CCK-8 testing of different structural scaffolds each time point. (**C**) The expression of osteogenesis-related genes on scaffolds, including OPN, BSP, OCN. (**D**) Simulation and (**E**) quantitative analysis of WSS for different structural scaffolds (*P < 0.05, **P < 0.01, ***P < 0.001).

The requirements of strength and porosity are fundamental for maintaining the structure at the defect site and supporting the growth of blood vessels and new bone. Permeability plays a crucial role in determining the ability of nutrient delivery and waste metabolism in porous structures. Meanwhile, appropriate permeability promotes tissue regeneration and enhances the implantation rate of the scaffold [35, 36]. The cubic structure was cut and simplified, and then, analyzed for permeability using COMSOL 6.1 (Figure 6A). The initial cube model generated by Rhino was cut into parts for simulation, due to the excessive memory requirements for simulation operations. Considering that the dimensional shrinkage rate after sintering is 30%, the cut model was scaled again to simulate the permeability of the support under the real size. The different sizes of IG scaffold were since the fusion unit needs to combine two-unit structures. In Darcy's Law, permeability is an inherent property of homogeneous porous scaffold and should be independent of size theoretically. Therefore, it is considered that the main influencing factor is the structural distribution. The differences in the calculation simulation diagram of WSS in Figure 7 were for the same reason.

The scaffold design aimed to mimic the dense outside and sparse inside of the femur with G inside and I outside. The smooth transition between the units could be seen in the SEM results (Supplementary Figure S3). As shown in Figure 6B, the designed scaffolds satisfied the permeability of human natural cancellous bone (0.5–5 × 10 ^−8^ m^2^) [37]. The highest pressure occurred at the inlet and gradually tended to zero in the outlet region, confirming the boundary conditions used in the model. The G exhibited the significantly highest permeability (1.55 × 10 ^−8^ m^2^) due to the high porosity, while the other structures showed basically lower than 0.5 × 10 ^−8^ m^2^. To reach the enhancement in strength and permeability of the scaffolds, the I and G units were integrated by sigmoid function. As shown in Figure 6B and C, the integrated structure (IG) possessed superior mechanical strength (5.8 ± 0.6 MPa) and improved permeability (0.97 × 10 ^−8^ m^2^). The design of the heterogeneous scaffolds was based on compressive strength and permeability properties combined with pore size, porosity and pore structures. Both P and D structure were not outstanding in terms of permeability and compressive strength, thus, were excluded from subsequent experiments.

### Cytocompatibility and osteogenesis of TPMS-based scaffolds

The biocompatibility of the scaffolds was evaluated by CCK-8 and live-dead staining experiments. As shown in Figure 7A, it could be found that the number of cells on the C structure was significantly less than that on the TPMS at 3 days, which attributed to the fact that the C structure restricted cell adhesion. On the contrary, there were adequate and continuous surfaces in the TPMS, which were more favorable for the adhesion and proliferation of cells. In addition, CCK-8 experiment was also performed and the results were shown in Figure 7B. Cell proliferation on the TPMS scaffolds was significantly better than that on the C structure, which suggested that the improvement of the permeability also boosted the proliferation of BMSCs. After being cultured on the scaffolds for 7 days, the expressions of osteogenesis-related genes were detected by RT-PCR. The results showed that BMSCs exhibited poor osteogenic differentiation on the C, with all osteogenesis-related genes down-regulating, while the I, G and IG all significantly up-regulated the expression osteogenesis-related genes (Figure 7C). Mechanical stimulation can make an effect on the differentiation of mesenchymal stem cells (MSCs), and the average WSS values for osteogenic differentiation of MSCs used in previous researches ranged from 0.1 to 10 mPa [33]. As shown in Figure 7D and E, there was higher stress at the intersection nodes of the C and at the narrower regions of the channels in the TPMS. Quantification of the WSS distribution demonstrated that the C had the largest high stress area, with 30.8% of the surface possessing a WSS greater than 10 mPa, and that excessive mechanical stimulation might not be suitable for osteogenic differentiation. In addition, it was observed that the TPMS scaffolds exhibited enhanced stress due to a sharp increase of the flow rate caused by a substantial change in the pore channel. The G scaffold exhibited superior permeability, while the IG reduced high stress area by integrating the G with lower WSS. Summarizing the above experiments, the prepared IG possessed more favorable mechanical strength, permeability and bioactivity compared with the C structure. As for the TPMS, the IG was optimized by a simple sigmoid function, making it well-suited for bone repair applications.

### Evaluation of *in vivo* repair of BG scaffolds

A model of rabbit femoral condylar defect was used to evaluate the bone repair effect of BG scaffolds. Combining the previous *in vitro* mineralization experiments and cellular experiments, 2Sr@BG was considered to possess excellent mineralizing activity, osteogenic and angiogenic activities. In terms of structure, the C structure was selected for comparison with the designed IG structure. Therefore, four groups of scaffolds (Blank, C, IG and Sr@IG) were designed for *in vivo* application. The surgical process was displayed in Supplementary Figure S4.

**Figure 8.**
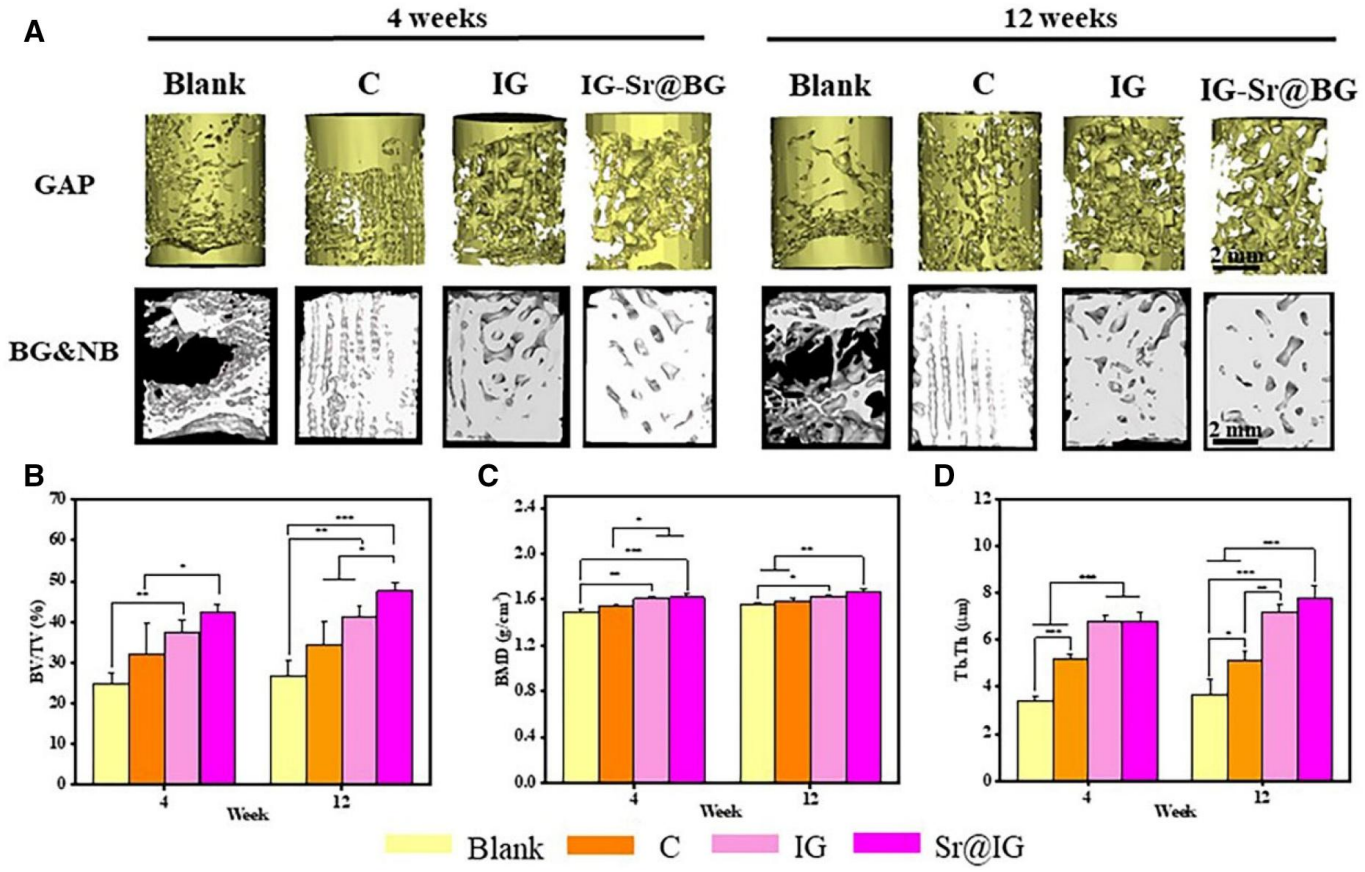
Micro-CT analysis of new bone production after 4 and12 weeks of scaffolds implantation. (**A**) Micro-CT images of scaffolds and new bone. Quantitative analysis of new bone formation by the index of (**B**) new bone volume/total volume (BV/TV), (**C**) bone mineral density to native bone (BMD) and (**D**) trabecular thickness (Tb.Th) (*P < 0.05, **P < 0.01, ***P < 0.001).

There was no apparent inflammation observe in all the scaffolds when being removed. The scaffolds were then subjected to Micro-CT reconstruction. The parts of BG and the new bone in the model with a minimum threshold of 1200 were extracted, since the density between BG scaffold and new bone was close, and cannot be clearly distinguished on reconstructed image of Micro-CT. However, it can be seen from the maximum cross-sectional view of the scaffold that the positions originally belonging to the pores of the BG scaffold have been filled with substances that might be new bone. The quantitative parameters of new bone volume/total volume (BV/TV), bone mineral density (BMD) and trabecular thickness (Tb.Th) were carried out through the CTAn software algorithm combined with histological verification to ensure data reliability. Take the new bone and scaffold as a whole, fewer voids implied a greater volume of bone and scaffold at the defect, and less structural clarity in the profile indicated more pronounced bone growth. A significant gap was presented in the blank group, suggesting that there was no obvious bone growth at 4 weeks or 12 weeks from Figure 8A. On the contrary, all the other groups showed obvious bone growth into the scaffold and reduction of the defect. The C structure showed less new bone formation than that in TPMS structure, with only a little bone in the lateral side. The TPMS structure showed pronounced growth of bone into the scaffolds from the scaffold section. New bone formed mainly from the lateral side because the void of the I was not evident on the outside of the scaffolds at 4 weeks. In addition, the doping of Sr demonstrated a better bone repair, with more new bone growing into the scaffolds. And the G inside the Sr@IG scaffold was less pronounced at 12 weeks. As shown in Figure 8B–D, the quantitative analyses of ROI (region of interest) by CTAn conformed this trend.

**Figure 9.**
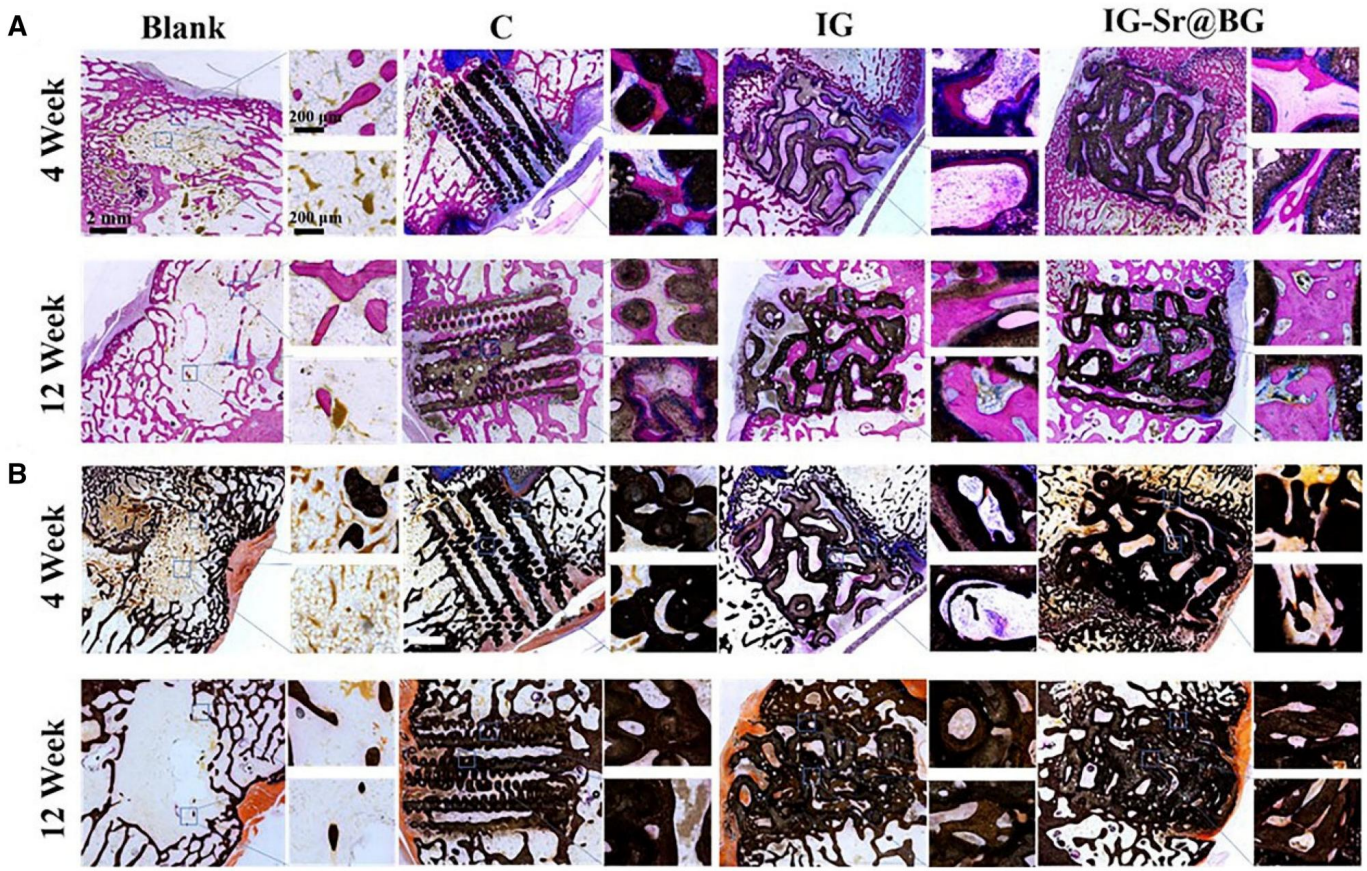
Histological analysis of the defect by (**A**) methylene blue/basic fuchsin staining and (**B**) Van kossa staining. The top right was a magnification of the defect-scaffold junction, and the bottom right was a magnification of the center of the defect or scaffold (×100).

Methylene Blue/Basic Fuchsin and Van Kossa staining were performed on hard tissue sections to visualize the growth of new bone into the scaffold (Figure 9). New bone formed mainly at two locations including the interface between the side of the scaffold and surrounding cancellous bone, and the interior of the scaffolds. Methylene blue is a basic dye that binds to anions in the DNA molecule to form a dark blue deposit. Basic fuchsin is a basic dye that can be used to stain collagen fibers, elastin fibers and nuclei of central nervous tissue, being able to stain them red accordingly. Thus, the nuclei of osteoblasts are dark blue or dark purple, and the extracellular matrix is light blue, and the new bone tissue is red after staining (Figure 9A). At 4 weeks and 12 weeks, the blank group exhibited only a small amount of discontinuous new bone production on the lateral side, and there was no new bone inside the defect. Similarly, there was only a small amount of new bone inside the scaffold in the C structure group at 4 weeks. The bone grew mainly around the struts, suggesting that the scaffold could support and guide the bone ingrowth to some extent. However, the distribution of new bone was not uniform and formed only in some of the struts. At 12 weeks, there was obviously more bone growth on the lateral and inside the scaffold. The bone on the surface of the IG scaffolds increased abundantly, and almost filled the interior and lateral sides. However, the interior of the scaffold near the surface of the defect lacked bone formation, which suggested that the bone failed to completely penetrate the scaffolds. Furthermore, there was more bone on the surface of the scaffolds compared to the IG group at 4 weeks and 12 weeks, indicating that the doping of Sr ion increased the formation of new bone. At 12 weeks, the bone penetrated the entire scaffold and showed the best osteogenic performance, indicating that the TPMS-based structure and the doping of Sr ion into the scaffolds jointly facilitated the osteogenic performance. In addition, Van Kossa was used to visualize calcium deposition. When a silver solution is applied to a slice containing insoluble calcium salts, the calcium is displaced by the silver and the silver salts are reduced to ferrous metallic silver in the presence of light. Calcium on bone tissue and scaffolds was stained dark brown and uncalcified bone was stained light brown or dark pink (Figure 9B). The trend of new bone formation between groups was consistent with the results of Methylene Blue/Basic Fuchsin staining.

## Discussion

BGs were widely used in the construction of bone tissue engineering scaffolds due to their high osteoconduction and osteoinductivity. However, the crystallization that occurs during the sintering limits its ability of molding and bioactivity. Therefore, 13-93BG with a larger sintering-working window becomes a suitable option. The doping of MgO and K_2_O has been shown to significantly reduce the crystallization tendency by increasing the content of network formers [28] and the mixed base effect [38], respectively. When the CaO (mol%) in the composition of 13-93 BG was replaced with SrO (mol%), the structure of the BG network did not change significantly and the biological activity was still maintained. In this work, there was no crystallization with the doping of Sr, while all Sr@BGs showed similar infrared peaks, indicating that there was no significant change in the structure.

*In vitro* mineralization experiments suggested that the dissolution rate of all ions was accelerated by the expansion of the BG network due to the increased Sr content [39]. The peak of the corresponding XRD shifted to a smaller degree due to the enlarged network (Supplementary Figure S1B), which could be verified by Bragg’s law that the spatial size between atoms of the glass network is inversely proportional to the diffraction angle [40]. However, it was found that the release of Ca^2+^ was inhibited as the introduction of Sr increased. Low levels of Sr@BGs (<5 mol%) were only able to rapidly consume Ca^2+^ after 3 days to form HAp. The possible reason was that the Sr-O bond is stronger than the Ca-O bond (351 kJ/mol for Ca-O and 389 kJ/mol for Sr-O), which made Sr^2+^ more difficult to release from the glass than Ca^2+^, thereby reducing the ability of Sr^2+^ to exchange with H^+^ in solution to promote the formation of apatite layers. In addition, the formation of HAp may start with the formation of Ca_8_(PO_4_)_6_H_2_·5H_2_O (OCP). When the Sr/Ca concentration in the environment is above a certain ratio, the nucleation site in OCP was replaced by abundant Sr^2+^, which occupied the activation site and hindered the nucleation [41]. Based on our experimental results, it appeared that Sr@BGs with Sr < 5 mol% remained biologically active and mineralized normally in 7 days. In addition, the released ions tended to act within a certain concentration range. The content of strontium ions in the human body is approximately 320 g, and mainly (above 99%) exists in the form of bones and hard tissues such as teeth [42]. The IG-Sr@BG scaffold with a diameter of 6 mm and a height of 12 mm was measured to be approximately 0.12 g, the total strontium content was 2.4 mg after being converted. Then, taking the release rate into account as well, the effect of strontium ion release from the scaffold on serum strontium concentration and the total strontium content throughout the body was very small. On the other hand, many studies have shown that low concentrations of strontium ions can also exert osteogenic effects on the body [43, 44]. Although the optimal concentration of Sr ion remains controversial, most studies have concluded that lower levels of Sr exhibited a positive effect (0.2-21 μg/mL) in bone restoration [32, 45]. Based on this fact, it was speculated that scaffolds that locally release strontium ions of low concentration behave osteogenic effects. In our present work, 2Sr@BG with a low Sr content (6.5 ppm) showed the best osteogenic activity. In addition, Vessel formation is also an aspect that has been explored. Previous studies showed that released Si and Sr increased the expression of angiogenesis-related genes (VEGF, Arg-1, CD31, etc.) with synergistic enhancement of angiogenesis. Sr ion played a major role in this process, which might be related to the stimulation of the PI3K/AKT signaling pathway in HUVECs and promoted angiogenesis [46, 47]. The Sr@BGs powders extracts exhibited a trend in promoting angiogenesis consistent with osteogenic differentiation. The expression of genes related to angiogenesis upregulated, and then, down-regulated with the doping of Sr content arising.

Previous studies have shown that DLP-printed bioceramic scaffolds with high porosity exhibited poor mechanical strength (0.33–5 MPa) [32, 48, 49]. On the one hand, the commonly used C structure displayed high stress concentration, while TPMS structures increased mechanical strength by dispersing stress [15, 49]. On the other hand, the lower solid content (<40 vol%) during DLP printing led to severe shrinkage of the scaffold [2] or premature crystallization [50]. 13-93 BG possessed a large sintering window has been verified based on our previous study [51], and the introduction of a small amount of Sr has no significantly effect the sintering behavior of the 2Sr@BG (Supplementary Figure S5). The crystallization-free BG scaffolds prepared with BG at 50 wt% (66 vol%) were used to enhance the mechanical property through structural design and material selection. TPMS is characterized by its ability to flexibly adjust the structure (curvature, period, etc.), as well as to achieve complex calculations such as booleaning and partitioning for desired construction [52]. Previous studies have found that a single design often failed to satisfy the needs, and heterogeneous TPMS were proposed for more complex applications in achieving improvements in mechanism and accurately mimicking the natural bone [53]. In this work, the differences in the mechanical strength and permeability of TPMS scaffolds were synthesized to meet the requirements of natural bone by integrating the G and the I units using a sigmoid function. Permeability is a critical parameter as it allows diffusion of nutrients and subsequent cell behaviors. Natural bone is composed of multiple pore scales, resulting in multiple levels of permeability ranging from 0.5 to 5 × 10 ^−8^ m^2^ [54]. Consistent permeability analyses were performed for each structure, and the results showed an order of G > IG > C > I > P > D. Except for the G and the integrated IG, the structures failed to meet the minimum requirement of permeability. In terms of the mechanical strength, the IG scaffolds were also significantly improved and could be applied to defects in cancellous bone. Another prominent feature of the TPMS is the connected curved surface in three-dimensional space, which enhances cell seeding and proliferation [15]. The C was composed of cylinders and cells grew only on smaller surfaces that were not continuous, which limited cell activity and osteogenic differentiation on the scaffold. In contrast, there was continuous space for cells to adhere and proliferate rapidly on the TPMS-based scaffolds compared to the C. Despite the greater surface area of the I, the lower permeability reduced cell proliferation. The IG scaffold showed comparable proliferation and significant upregulation expression of osteogenic genes compare to G at 7 days. In addition, WSS is a mimetic predictor of the deposition and differentiation behavior of MSCs on scaffolds stimulated by mechanical stress. However, various WSS distribution can lead to inhomogeneous cell differentiation within the scaffolds [33]. MSCs tend to differentiate into chondrocytes at a WSS of 10–30 mPa and into osteoblasts at a WSS below 10 mPa [55]. There was a lower percentage of high WSS area which may be beneficial for osteogenic differentiation.

The excellent osteogenesis of the designed Sr@IG scaffold was further validated in a rabbit femoral condylar defect model. The IG scaffolds showed a significantly increased number of continuous new bone wrapped around the interface of the scaffolds compared with the C structure at early and later stage. The Sr@IG scaffolds exhibited increased bone formation, with bone extending throughout the entire scaffolds. In addition, none of the scaffolds showed obvious fragmentation and degradation, indicating that the porous scaffolds could provide adequate mechanical support and promotes bone tissue regeneration.

## Conclusion

In conclusion, TPMS scaffolds with favorable mechanical strength and high porosity were manufactured by DLP printing. The design of the structure involved TPMS structures, integrating high-strength I unit and high-permeability G unit to improve the performances of the scaffold. The compressive strength test showed that the IG scaffold displayed a compressive strength of 5.8 ± 0.6 MPa. Permeability analysis also revealed that the IG scaffold met the needs of human bone permeability (0.97 × 1 0 ^−8^ m^2^) adequately. In addition, the IG structure exhibited superior bioactivity compared to the C structure in terms of cell viability, proliferation and osteogenic differentiation. *In vivo* experiment verified that the IG scaffold reflected significantly more new bone generation compared to the C structure group, and the doping of Sr in Sr@IG effectively promoted the growth of new bone into the scaffolds with optimal osteogenesis. These results suggested that the ionic doping and structural design of the scaffolds both facilitated the osteogenic performance, and also provided a promising solution for the customized fabrication of artificial bone scaffolds with specific structure and enhanced bioactivity.

## Supplementary Material

rbaf053_Supplementary_Data
