# Supplementary material for: Three-dimensional-printed triply periodic minimal surface scaffolds via digital light processing for enhanced osteogenesis
Source: Regen Biomater. 2025 Jun 5;12:rbaf053. doi: 10.1093/rb/rbaf053 (PMC12596146; doi:10.1093/rb/rbaf053)
Supplement: rbaf053_Supplementary_Data [file rbaf053_supplementary_data.docx]

# Surpporting information

**Three-dimensional-printed triply periodic minimal surface scaffolds via digital light processing for enhanced osteogenesis**

# Weilong Zou ^a †^, Xiyuan Han ^b †^, Qiyuan Dai ^b^, Zequ Lin ^b^, Qingtao Li ^c^, Zilin Li ^b^, Xinrong Xu ^b^, Xinying Chen ^a^, Huichang Gao ^c*^, Xiaodong Cao ^b,d,e*^

^a^ Department of Spinal surgery, Heyuan People's Hospital, Heyuan, 517000, PR China

^b^ School of Materials Science and Engineering, South China University of Technology, Guangzhou, 510641, PR China

^c^ School of Medicine, South China University of Technology, Guangzhou, 510006, PR China

^d^ National Engineering Research Centre for Tissue Restoration and Reconstruction, South China University of Technology, Guangzhou, 510006, PR China

^e^ Key Laboratory of Biomedical Engineering of Guangdong Province, South China University of Technology, Guangzhou, 510006, PR China

†Weilong Zou and Xiyuan Han are equally contributed to this work.

*Corresponding author.

Huichang Gao, E-mail addresses: mchcgao@scut.edu.cn

Xiaodong Cao, E-mail addresses: [caoxd@scut.edu.cn](mailto:caoxd@scut.edu.cn)

**Table S1.** Compositions of the Sr@BGs in mol%.

| Sample group | SiO_2_ | P_2_O_5_ | K_2_O | Na_2_O | MgO | CaO | SrO |
| --- | --- | --- | --- | --- | --- | --- | --- |
| 0Sr@BG | 54 | 2 | 8 | 6 | 8 | 22 | 0 |
| 2Sr@BG | 54 | 2 | 8 | 6 | 8 | 20 | 2 |
| 5Sr@BG | 54 | 2 | 8 | 6 | 8 | 17 | 5 |
| 10Sr@BG | 54 | 2 | 8 | 6 | 8 | 12 | 10 |
| 22Sr@BG | 54 | 2 | 8 | 6 | 8 | 0 | 22 |

**Table S2.** Components of SBF

| Oder | Reagent | Amount for 1L SBF |
| --- | --- | --- |
| 1 | NaCl | 7.996 g |
| 2 | NaHCO_3_ | 0.35 g |
| 3 | KCl | 0.224 g |
| 4 | K_2_HPO_4_·3H_2_O | 0.228 g |
| 5 | MgCl_2_·6H_2_O | 0.305 g |
| 6 | HCl (1mol/L) | 40 mL |
| 7 | CaCl_2_ | 0.278 g |
| 8 | Na_2_SO_4_ | 0.074 g |
| 9 | NH_2_C(CH_2_OH)_3_ | 6.057 g |

**Table S3.** Primer sequences for all RT-PCR experiments.

| **HUVEC genes** | **Forward primer (5’-3’)** | **Reverse primer (5’-3’)** |
| --- | --- | --- |
| GAPDH | GATTTGGTCGTATTGGGCG | CTGGAAGATGGTGATGG |
| HIF-1α | CAGAAGATACAAGTAGCCTC | CTGCTGGAATACTGTAACTG |
| eNOS | CTCCAGCCCCGGTACTACTC | TTAGCCACGTGGAGCAGACT |
| VEGF | TGCGGATCAAACCTCACCA | CAGGGATTTTTCTTGTCTTGCT |
| KDR | GGCATGGGGTCTGTTCTGAA | TTGGCCAGGAGACACGTAAC |
| **BMSCs genes** | **Forward primer (5’-3’)** | **Reverse primer (5’-3’)** |
| GAPDH | GTTCCTACCCCCAATGTGTCCC | TAGCCCAAGATGCCCTTCAGT |
| OCN | AGCAGCTTGGCCCAGACCTA | TAGCGCCGGAGTCTGTTCACTAC |
| OPN | TGCAAACACCGTTGTAACCAAAAGC | TGCAGTGGCCGTTTGCATTTCT |
| BSP | AGACAACGGAGAAGAAGCCG | TCTCCCCCATACTCAACGGT |
| Col I | ATGCCGCGACCTCAAGATG | TGAGGCACAGACGGCTGAGTA |

**
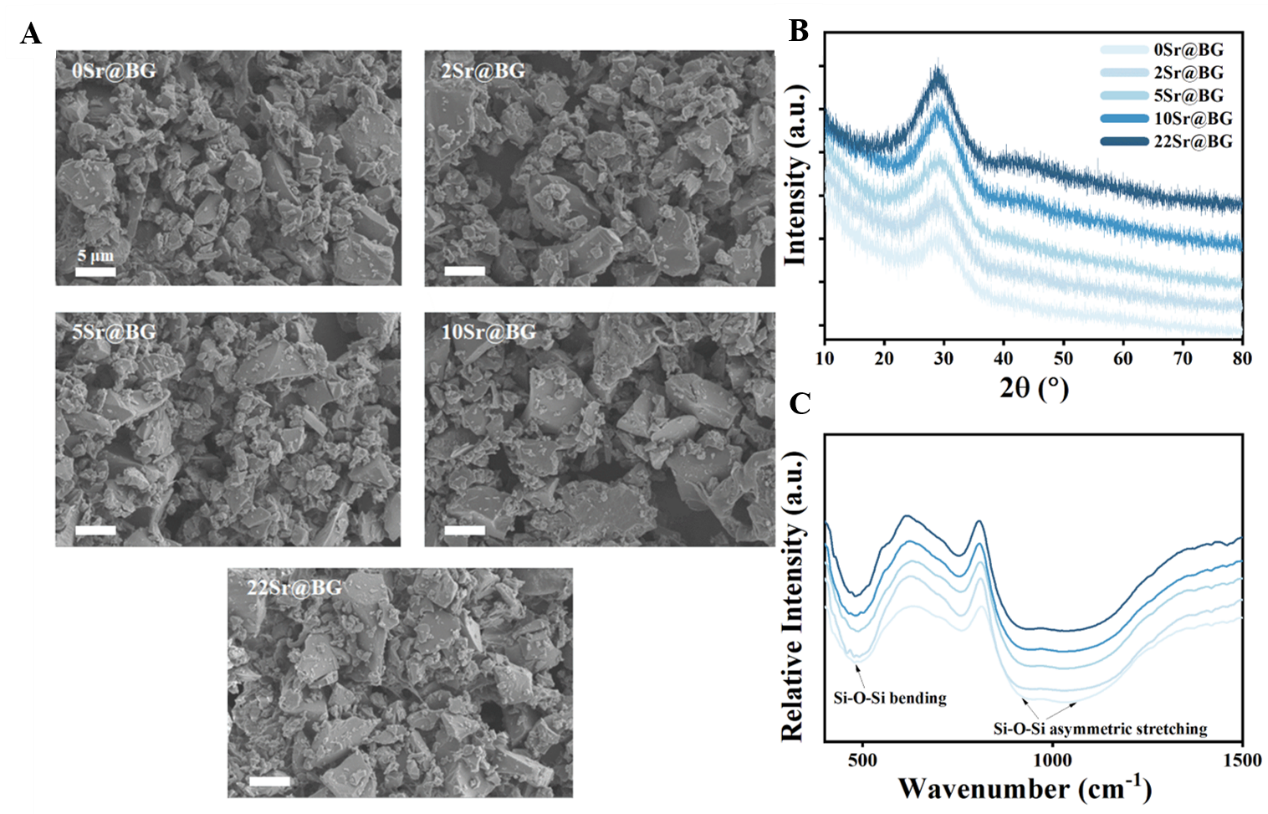
**

Figure S1. Morphology and structure of Sr@BGs powder with different Sr content. (A) SEM images of Sr@BGs powder with different Sr content. (B) XRD characterization of each Sr@BGs powder with different Sr content. (C) FTIR characterization of each Sr@BGs powder with different Sr content.


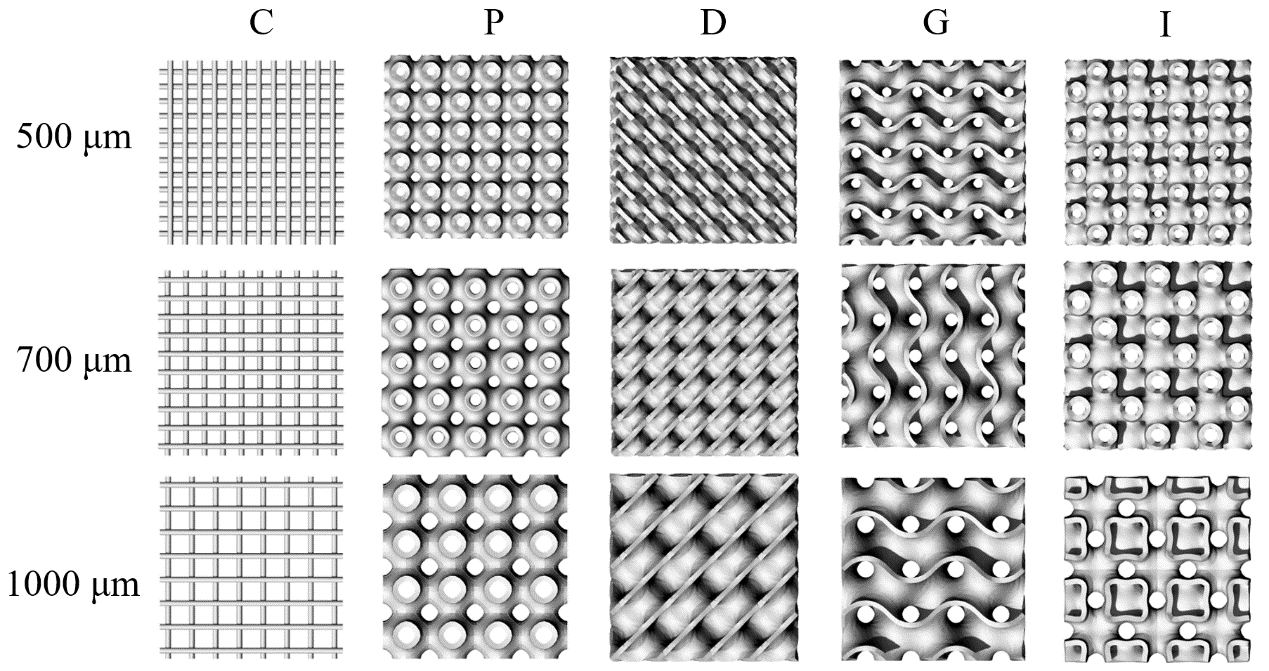


Figure S2. TPMS structures with different pore sizes (1000 μm, 700 μm, 500 μm) in Rhino 7.


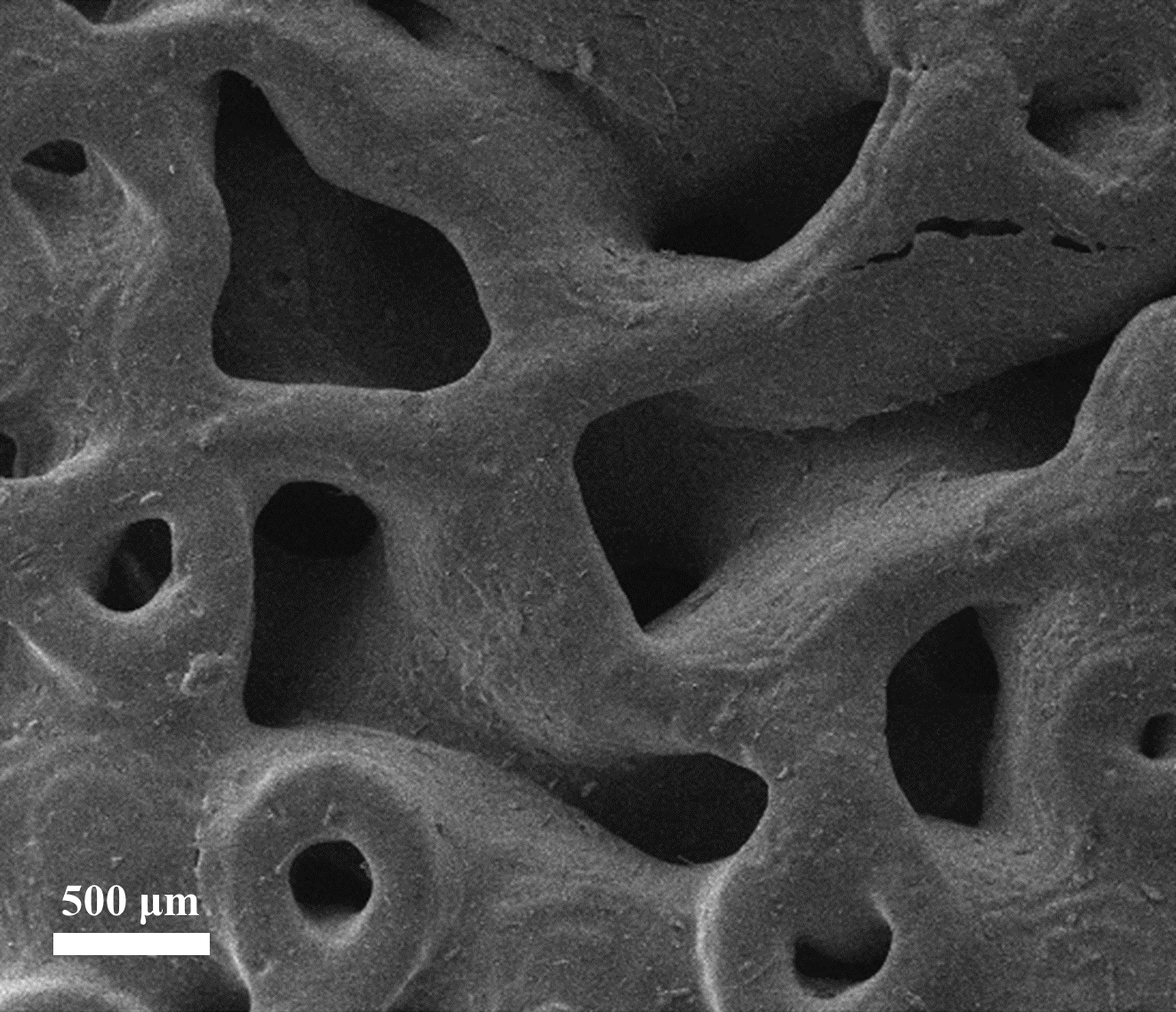


Figure S3. SEM image of IG scaffold.


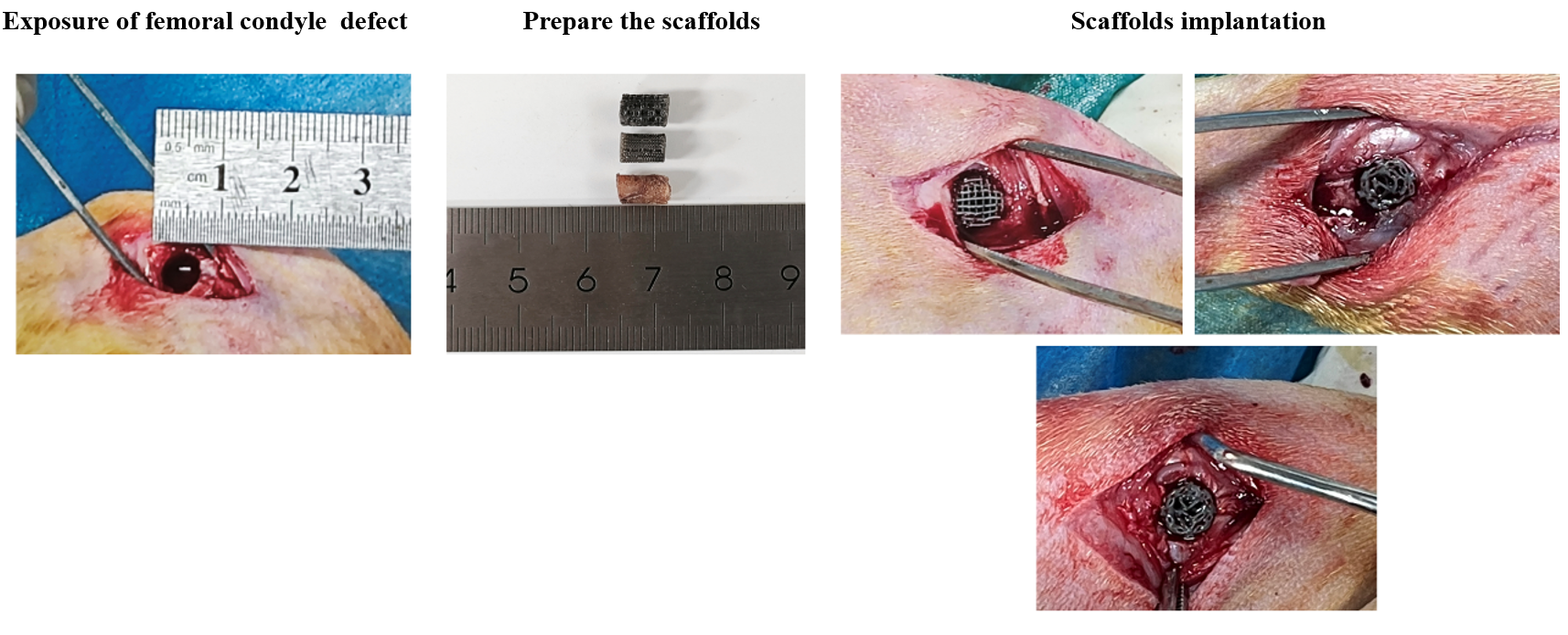


Figure S4. Image recordings during the operation.


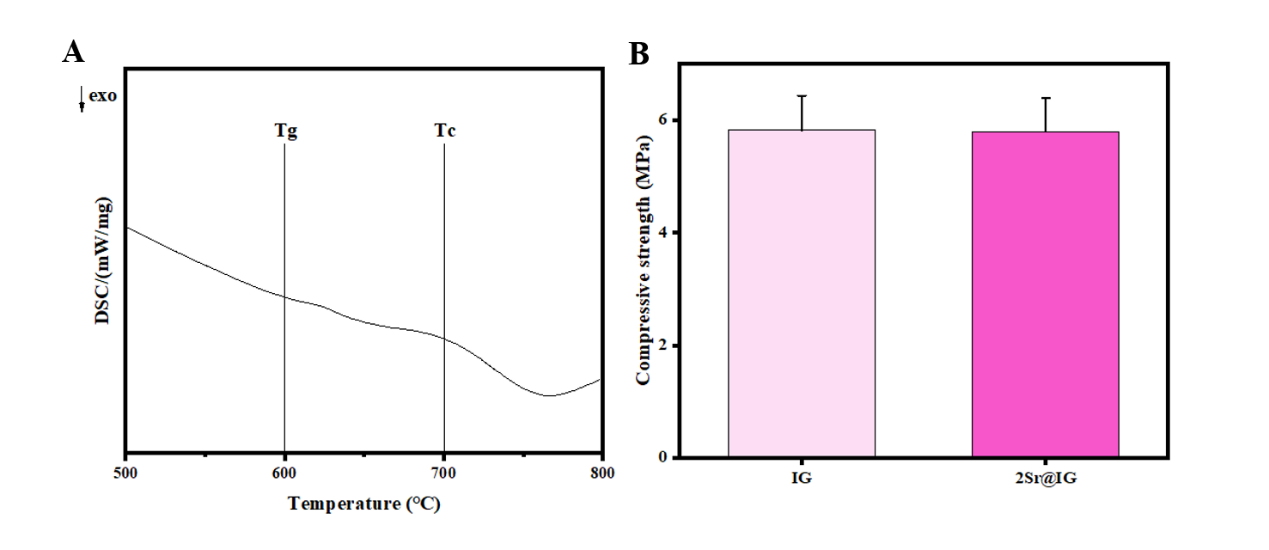


Figure S5. The sintering ability of 2Sr@BG powder and the property of porous 2r@BG scaffolds. (A) Differential Scanning Calorimetry (DSC) analysis of 2SrBG powder. Tg is Glass transition temperature and Tc is crystallization temperature. (B) Compressive strength of IG scaffold with or without Sr.


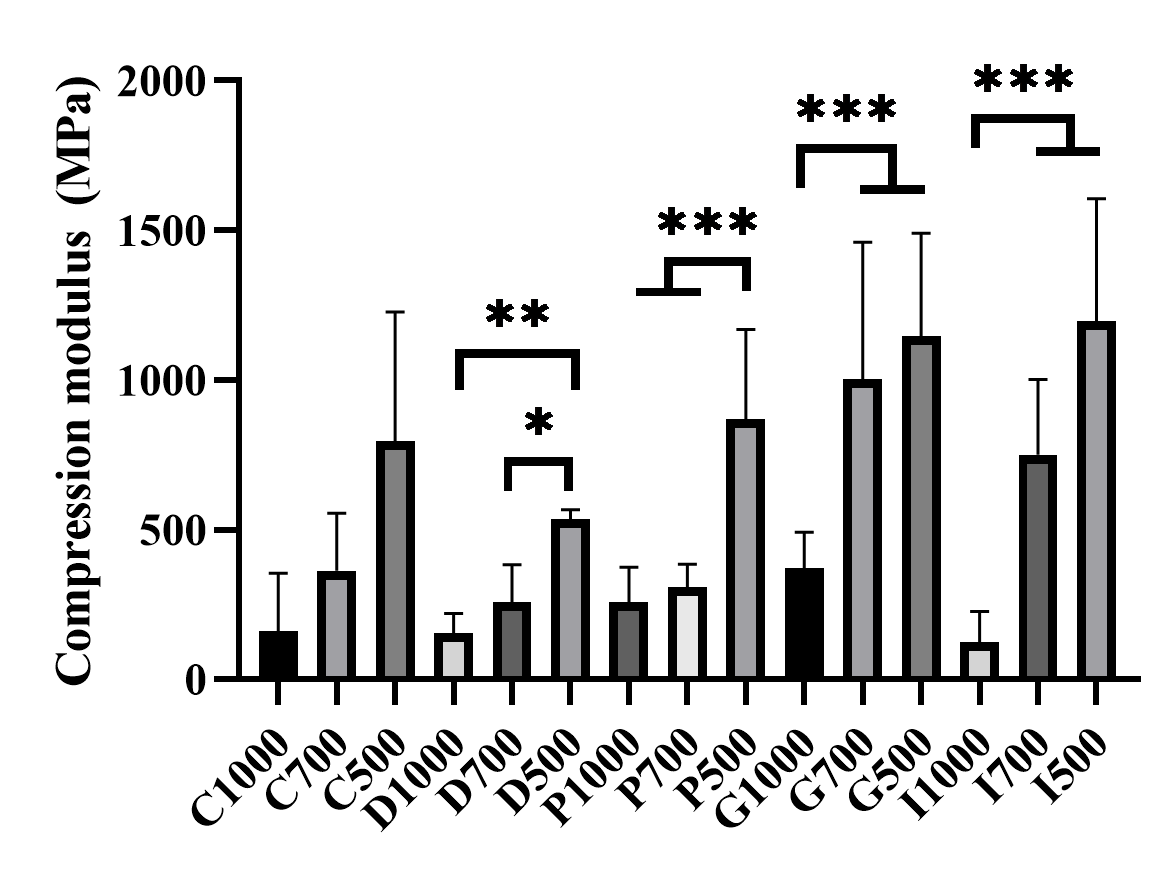


Figure S6. Compressive modulus of different structural BG scaffolds with different pore sizes (1000, 700, 500 μm). (*P < 0.05, **P < 0.01, ***P < 0.001).
